# Cardiovascular medication utilization and adherence among adults living in rural and urban areas: a systematic review and meta-analysis

**DOI:** 10.1186/1471-2458-14-544

**Published:** 2014-06-02

**Authors:** Gaetanne K Murphy, Finlay A McAlister, Daniala L Weir, Lisa Tjosvold, Dean T Eurich

**Affiliations:** 1 2-040 Li Ka Shing Center for Health Research Innovation, School of Public Health, University of Alberta, Edmonton, AB T6G 2E1, Canada; 2 Division of General Internal Medicine, Department of Medicine, Medicine and Dentistry, University of Alberta, Edmonton, Canada; 3 Alliance for Canadian Health Outcomes Research in Diabetes, School of Public Health, University of Alberta, Edmonton, Canada

**Keywords:** Cardiovascular diseases, Rural population, Urban population, Medication adherence, Drug utilization

## Abstract

**Background:**

Rural residents face numerous barriers to healthcare access and studies suggest poorer health outcomes for rural patients. Therefore we undertook a systematic review to determine if cardiovascular medication utilization and adherence patterns differ for rural versus urban patients.

**Methods:**

A comprehensive search of major electronic datasets was undertaken for controlled clinical trials and observational studies comparing utilization or adherence to cardiovascular medications in rural versus urban adults with cardiovascular disease or diabetes. Two reviewers independently identified citations, extracted data, and evaluated quality using the STROBE checklist. Risk estimates were abstracted and pooled where appropriate using random effects models. Methods and reporting were in accordance with MOOSE guidelines.

**Results:**

Fifty-one studies were included of fair to good quality (median STROBE score 17.5). Although pooled unadjusted analyses suggested that patients in rural areas were less likely to receive evidence-based cardiovascular medications (23 studies, OR 0.88, 95% CI 0.79, 0.98), pooled data from 21 studies adjusted for potential confounders indicated no rural–urban differences (adjusted OR 1.02, 95% CI 0.91, 1.13). The high heterogeneity observed (I^2^ = 97%) was partially explained by treatment setting (hospital, ambulatory care, or community-based sample), age, and disease. Adherence did not differ between urban versus rural patients (3 studies, OR 0.94, 95% CI 0.39, 2.27, I^2^ = 91%).

**Conclusions:**

We found no consistent differences in rates of cardiovascular medication utilization or adherence among adults with cardiovascular disease or diabetes living in rural versus urban settings. Higher quality evidence is needed to determine if differences truly exist between urban and rural patients in the use of, and adherence to, evidence-based medications.

## Background

Rural and urban communities have distinct characteristics in terms of demographics, social, and physical environments, and may vary in access to healthcare facilities and services. Rural residents tend to be older and are more likely to be obese, have less education, and lower income than urban residents [1-6]. Rural populations also have a higher prevalence of chronic conditions such as diabetes and heart disease [1,7-9]. Some studies have shown worse health outcomes among rural populations, including a higher risk of cardiovascular-related morbidity and mortality compared to urban populations [3,4,10-13]. For example, in Canada, mortality from circulatory diseases is higher in rural than urban communities, as is the risk of heart failure-related mortality, hospitalization and emergency department visits [4,10,11]. Similarly, in the UK a higher risk of in-hospital death due to ischemic heart disease has been observed among rural residents [12], and in Australia, mortality due to six major chronic diseases consistently increased in areas that were increasingly remote [13]. Collectively, these characteristics suggest increased healthcare needs for those living in rural communities.

However, rural residents report several barriers to accessing healthcare including transportation difficulties and distance to care, social isolation, financial constraints, limited health care facilities (hospitals and pharmacies), physician shortages, and lack of access to specialist care [14-19]. Indeed, in the US, rural areas contain 19% of the population but only 11% of the physician workforce [15], and the ratio of specialists per population consistently declines as locations become smaller and more remote [15,17]. The lack of access to primary care physicians, specialists, or health care facilities has been postulated to result in decreased prescribing of evidence based medications. However, a previous systematic review found no clear rural–urban difference in the prevalence or intensity of prescription drug use in older adults – although that review included a wide variety of health conditions and medications [20]. It is possible that important differences may exist for certain disease states or medication conditions. As a result, we conducted a systematic review that evaluated whether cardiovascular-related medication utilization and adherence patterns differ for rural versus urban adults with cardiovascular disease or diabetes. These two disease states were selected as they affect a large number of patients, are associated with high morbidity and mortality, often require multiple medications to manage, and outcomes are known to be different between rural and urban patients [21].

## Methods

### Inclusion and exclusion criteria

Controlled clinical trials or observational studies were included if they enrolled adults with established cardiovascular disease (atrial fibrillation, hypertension, heart failure, coronary artery disease) or diabetes, and reported cardiovascular medication use or adherence patterns for patients living in rural versus urban communities. Medications of interest included acetylsalicylic acid (ASA), antithrombotic, anticoagulant, antihypertensive (including angiotensin converting enzyme inhibitors (ACEI) and angiotensin receptor blockers (ARB)), or lipid lowering agents. The research question, inclusion and exclusion criteria, and review methods were outlined in a protocol developed a priori according to the PRISMA guidelines [22].

Since the definition of rural and urban varied substantially between studies, we a priori defined populations described as urban, city dwelling, or metropolitan in the primary publication as urban. Conversely, rural descriptors included town, village, country dwelling, non-metropolitan or remote communities. Any definition of adherence or persistence used in primary studies was accepted. Only full text, peer reviewed articles, were included. Studies evaluating the use of medications for acute management, such as during hospitalization, were excluded, as were studies conducted in developing countries where management approaches may be substantially different. The populations of interest were those with established cardiovascular disease or diabetes, in whom several evidence-based medications are recommended for use. Two researchers (GKM, DLW) independently screened all studies and extracted all data using pre-defined forms and definitions, and disagreements were resolved through discussion, or by a third researcher (DTE).

### Literature search strategy

A comprehensive search strategy implemented by a research librarian was done in April 2012 in the following electronic databases: MEDLINE®, PubMed, Embase, International Pharmaceutical Abstracts, CINAHL, and Web of Science® and reference lists of included articles were also manually searched. Previously identified included studies were searched in Scopus to gather additional subject headings. No language, study design or date restrictions were applied. The MEDLINE® search strategy is listed in (Additional file 1: Table S1).

### Data extraction and quality assessment

Studies were evaluated for bias, and the STROBE checklist was used to assess the quality of reporting [23]. Study authors were contacted for missing information on rural–urban comparisons, and in two cases additional data were provided (unpublished data V. Maio 2012, I. Carey 2011). Both unadjusted and adjusted data were abstracted or calculated where possible [24]. If more than one adjusted analysis was reported, the analysis that adjusted for the most confounders was extracted, and medication use data for patients without contraindications to treatment were preferred over populations that may have included patients who were not eligible for a specific therapy. Where possible, studies reporting multiple rural or urban populations were combined.

### Data analysis methods

To summarize the effects of rural and urban location on medication utilization or adherence both unadjusted and adjusted pooled effects were calculated. As we expected heterogeneity between studies, we pooled effect estimates using a random effects model with inverse variance weighting and Review Manager 5.1 software [25]. Heterogeneity was assessed using the I^2^ statistic with an I^2^ statistic >50% being considered as moderate heterogeneity. There was no a priori degree of heterogeneity that precluded pooling. For studies reporting multiple outcomes within the same cohort [e.g., % receiving beta-blockers (BB) and % receiving an ACEI], a pooled estimate of the odds of treatment were calculated using methods recommended by Borgenstein et al. [26] that accounts for the fact that patients within each outcome are not mutually exclusive (i.e., a patient may have received both a BB and an ACEI). Since the correlation of outcomes is unknown, we used a moderate correlation of 0.5 with sensitivity analyses using 0.25, 0.75 or 1 and found there was little impact on the results. Subgroup analyses were further conducted to explore the robustness of our results and potential sources of heterogeneity. Studies reporting data not suitable for meta-analysis (e.g. outcomes other than OR, or with missing data) were summarized narratively. Publication bias was assessed using funnel plots and Egger’s test.

## Results

**Figure 1:**
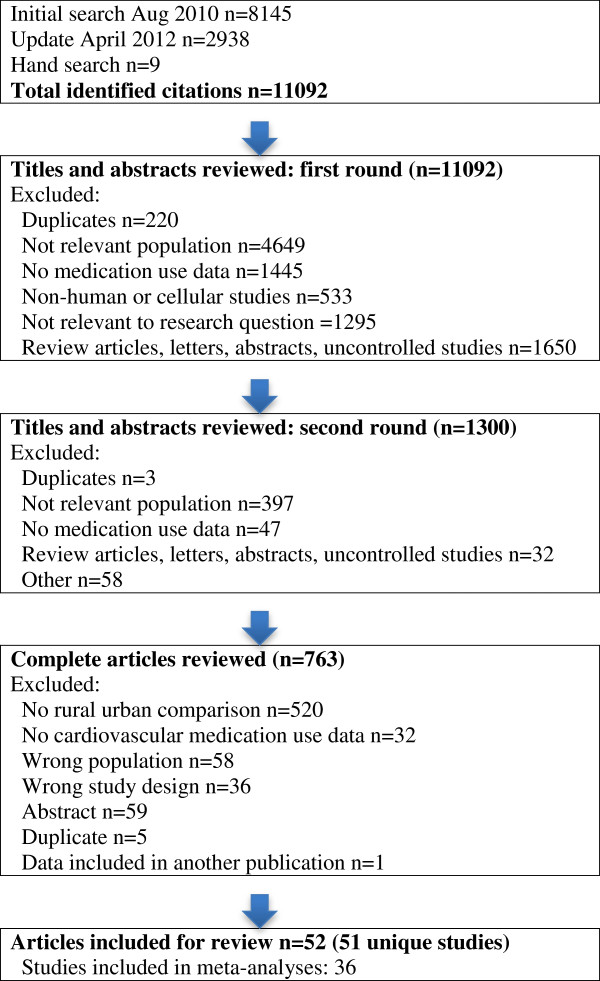
Flow chart of systematic search.

A total of 11092 citations were identified in the literature search and 51 unique studies (described in 52 publications), met the inclusion criteria (Figure 1) [2,8,9,27-75]. Fifteen studies were cohort studies and 36 were cross sectional or repeat cross sectional studies (Additional file 1: Table S2). The included studies were published over a 21-year time span (1990 to 2011) and had quality scores based on the STROBE checklist that ranged from 8.5 to 21 (median 17.5) out of a total of 22 possible items (0.5 points given for partial reporting). Two reports provided data on the same study [44,48], and six studies included data for more than one patient cohort [43,49,54,66,71,72]. Thus, 58 patient populations (or cohorts) were included in our study. Two studies were in a language other than English and were translated using on-line resources and local expertise [37,70]. There was good agreement between reviewers on study selection (kappa 0.82, 95% confidence interval [CI] 0.75 to 0.90).

Patients were selected from a hospital setting in 12 studies, from ambulatory care practices in 17 studies, and in 22 studies, patients were selected from population-based or community-dwelling samples. Exploring rural–urban differences was the primary objective of 18 studies (35%) [2,27,28,30,31,35,36,39,43,44,49,51,54,59,68,69,71,73]. Seven studies reported medication adherence (the proportion of doses taken as prescribed over a specific time period) or persistence (the length of continuous treatment) [76], and 47 reported outcomes related to medication utilization. Among the studies, there were substantial variations in how medication utilization or adherence to medications was assessed. Overall, 16 studies included patient self-report, 31 studies included data from medical records or registries, and 5 studies were conducted using administrative databases. Nineteen studies reported crude utilization or adherence outcome data only [8,28,35-37,39,40,50,51,53,54,63,64,67,68,71,73-75].

Included studies varied in their characteristics (Additional file 1: Table S2). Studies ranged in sample size from 32 to approximately one million and were conducted in a range of areas including the US (30 studies), Europe (14), Canada (5) and Australia (2). Patient populations included those with acute myocardial infarction or coronary artery disease (18 studies), hypertension (16), diabetes (8), chronic heart failure (6), atrial fibrillation (5), or mixed cardiovascular disease populations (5). The average age of study participants ranged from 42 to 80 years, and 28% to 63% were female.

### Medication utilization

**Figure 2:**
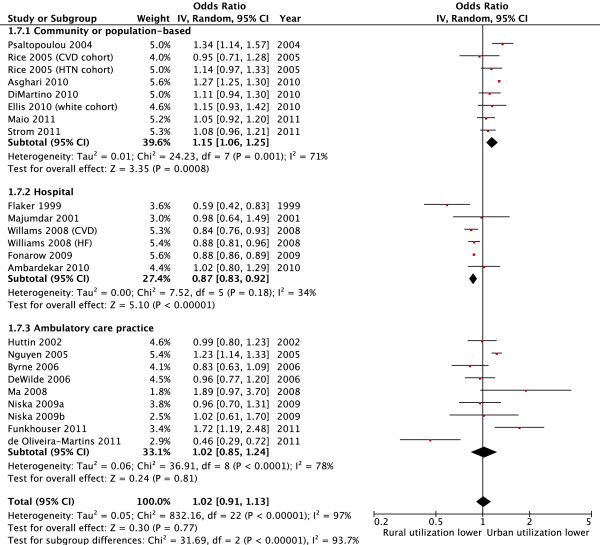
Use of cardiovascular drugs by rural versus urban patients, stratified by study setting (adjusted analysis).

**Figure 3:**
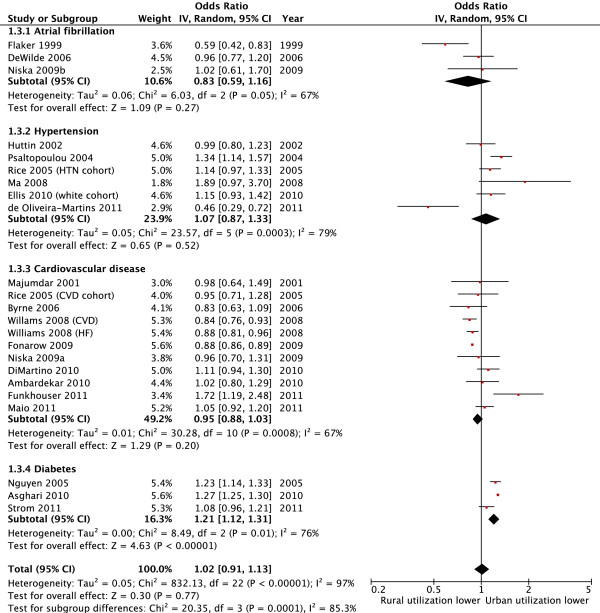
Use of cardiovascular drugs by rural versus urban patients, stratified by disease (adjusted analysis).

Forty seven (92%) studies [2,8,9,27-36,38-47,49,51-69,71-73,75] evaluated cardiovascular medication utilization with 20 (39%) studies specifically evaluating utilization of ASA or other anti-thrombotic agents, 34 (67%) evaluating antihypertensive use, and 11 (22%) evaluating the use of lipid lowering agents (Additional file 1: Table S3). Substantial variation in the use of cardiovascular medications was observed between studies and between rural versus urban sub-populations within each study. Indeed, the absolute difference in the utilization of cardiovascular medications ranged from -46% to +4% in rural versus urban patients for ASA or other anti-thrombotic drugs, -37% to +25% for antihypertensive drugs, and -45% to +8% for lipid lowering agents. Of the 47 studies that evaluated cardiovascular medication use, sufficient data for pooling were available in 34 studies (39 separate cohorts as two cohorts were included in three studies [43,66,72] and three cohorts in one study [71]). In the unadjusted pooled analyses (23 studies) [27-29,35,36,38,39,41,43,44,46,47,51,53,55-58,64,68,71,73,75], patients in rural areas with cardiovascular disease or diabetes were less likely to receive evidence-based cardiovascular drug therapy compared to urban residents (pooled unadjusted OR 0.88, 95% CI 0.79, 0.98, p = 0.02; I^2^ = 97%) (Table 1). However, among the 21 studies that adjusted for potential confounders [9,27,29,34,38,41-45,47,52,55,56,58,60-62,66,69,72] pooled analysis indicated no statistically significant difference between rural and urban patients in the use of cardiovascular medications (pooled adjusted OR 1.02, 95% CI 0.91 to 1.13, p = 0.77, I^2^ = 97%) (Figure 2). In both analyses there was substantial heterogeneity between studies. Although numerous subgroup analyses by setting, drug class, disease, age, country, and publication year were undertaken, similar results were observed and these factors only partially explained some of the variation between studies (Table 1; Figures 2 and 3). Moreover, when studies were categorized according to how the outcome was assessed (patient self-report, medical chart review, or administrative data), similar results were observed (Table 1). Additional analyses by study quality, for countries with universal health care systems, and for specific drugs, such as ACEI/ARB, also found similar findings (Additional file 1: Table S4). Among studies reporting data not suitable for meta-analysis, the findings were also consistent in that there was no clear trend towards a reduction or increase in cardiovascular medication utilization between rural and urban patients [2,8,30-33,40,49,54,59,63,65,67].

**Table 1:** Meta-analysis of medication utilization and adherence outcomes

**Analysis**	N cohorts*	**Rural/urban OR of treatment (95% CI)**	**I**^**2 **^**(%)**	**Subgroup difference**
**Cardiovascular medication utilization**				
Overall - unadjusted	26	0.88 (0.79, 0.98)	97	NA
Overall - adjusted	23	1.02 (0.91, 1.13)	97	NA
**Subgroup analysis – adjusted**				
*Setting*				
Community or population-based	8	1.15 (1.06, 1.25)	71	P < 0.0001
Hospital	6	0.87 (0.83, 0.92)	34	
Ambulatory care practice	9	1.02 (0.85, 1.24)	78	
*Drug class*				
Antithrombotic or anticoagulant	9	1.00 (0.88, 1.14)	88	P = 0.62
Antihypertensive	16	1.03 (0.90, 1.17)	92	
Lipid lowering agent	4	0.94 (0.83, 1.07)	37	
*Disease*				
Atrial fibrillation	3	0.83 (0.59, 1.16)	67	P = 0.0001
Cardiovascular disease	11	0.95 (0.88, 1.03)	67	
Hypertension	6	1.07 (0.87, 1.33)	79	
Diabetes	3	1.21 (1.12, 1.31)	76	
*Age (study mean or median)*				
<65 years	8	1.15 (1.04, 1.26)	80	P = 0.005
≥65 years	12	0.98 (0.88, 1.08)	70	
Not reported	3	0.89 (0.77, 1.02)	64	
*Country*				
US	18	1.01 (0.92, 1.10)	87	P = 0.67
Non-US	5	1.05 (0.88, 1.26)	88	
*Publication Year*				
1999-2005	7	1.06 (0.91, 1.22)	76	P = 0.66
2006-2011	16	1.01 (0.88, 1.15)	98	
*Data source*				
Administrative data	2	1.17 (0.97, 1.41)	87	P = 0.19
Medical record	14	0.97 (0.87, 1.08)	86	
Patient self-report	7	1.07 (0.93, 1.22)	72	
**Cardiovascular medication adherence**				
	**N**	**Rural/urban OR of treatment (95% CI)**	**I**^**2 **^**(%)**	**Subgroup difference**
Overall - unadjusted	4	0.94 (0.39, 2.27)	91	NA

*Data from 23 and 21 studies were included in the unadjusted and adjusted analyses, respectively: Ellis [43], Rice [66], Wan [71], and Williams [72] provided data for more than one cohort. CI = confidence interval; NA = not applicable; OR = odds ratio.

Publication bias was assessed by visually examining funnel plots and no obvious asymmetry was noted (Egger’s p value = 0.98).

### Medication adherence or persistence

Six (12%) studies [29,37,46,70,73,74] evaluated cardiovascular medication adherence and two (4%) studies [46,50] evaluated medication persistence. Adherence was measured as the percentage of doses taken [73], the proportion of patients with a medication possession ratio ≥0.8 [46], and was undefined in three studies [37,70,74]. Persistence was measured as the proportion of patients remaining on the same [46,50] or any treatment [46] at the end of the follow up. In five studies, the drugs evaluated were antihypertensive agents, one study evaluated heart failure medications [73], and one assessed ASA or ACEI/ARB adherence [29].

Cardiovascular medication adherence or persistence findings were inconsistent across studies (Additional file 1: Table S3). The absolute difference in proportion adherent or persistent with medications ranged from -41% to +8% for rural versus urban patients. The odds of treatment persistence were significantly higher in rural versus urban patients in one report (adjusted OR 1.28, 95% CI 1.25, 1.32) [46], but was not statistically different in a second study (OR 0.96, 95% CI 0.55, 1.67) [50], Medication adherence data from three studies (4 cohorts) were pooled and showed no statistically significant difference between rural and urban patients (unadjusted OR 0.94, 95% CI 0.39, 2.27, p = 0.89, I^2^ = 91%) Table 1[29,37,70]. In two other reports with data not suitable for meta-analysis, medication adherence was significantly higher in one study (92% versus 83% of doses taken, p = 0.01) [73], and significantly lower in the other (10% versus 17% of patients compliant, p < 0.01) [74], for rural versus urban patients, based on unadjusted data. The three studies reporting treatment adherence adjusted for confounders reported adherence to be significantly higher in rural patients (OR medication possession ratio ≥0.8: antihypertensive agents 1.2, 95% CI 1.1, 1.3; ASA 1.14, 95% CI 1.10, 1.18; ACEI/ARB 1.18 95% CI 1.14, 1.23) or rural men (OR adherent 4.0, 95% CI 1.1, 13.9) [29,46,70].

## Discussion

Our systematic review of the literature found that rural patients were 12% less likely to receive cardiovascular medications than urban patients in unadjusted analyses; however, pooling of data adjusted for patient, practitioner or other factors revealed no differences in the proportions of rural and urban patients receiving therapy. This suggests that differences in these characteristics between rural and urban residents are largely responsible for the discrepancies in medication use observed and is consistent with previous studies showing important differences in the demographics, health behaviors, and overall health of people living in rural and urban areas [1,3,4,7]. In this review, many of the included reports provided little data on the demographics or comorbidities of the rural and urban patient groups, which hindered our ability to assess the similarity of these populations. As a result, it is difficult to draw conclusions from those studies that reported only unadjusted rural–urban comparisons.

When medication utilization data were pooled, substantial between-study heterogeneity was observed (I^2^ = 97%). Some of this variability could be explained by differences in the setting (hospital, ambulatory care practice or community-based sample), age, and disease state. While most studies adjusted for some clinical characteristics, only some controlled for socioeconomic factors that could also have impacted medication use. Indeed only fourteen studies [27,32,34,42,43,45,47,52,57,58,61,62,66,69] reported adjustment for health insurance and previous studies have shown patients with a chronic condition who lack medication insurance are less likely to take medications or frequently skip doses due to cost [52,66,77].

Similarly, we found no consistent relationship between rural residence and cardiovascular medication adherence or persistence rates based on unadjusted data. The adjusted analysis suggested higher adherence and persistence for rural residents, although there were few studies reporting these outcomes. Rural–urban differences in other health behaviors, such as smoking, exercise, and consumption of fruits and vegetables have been reported [4], and considering the link between adherence and positive health outcomes [78], further study is warranted.

Although we conducted an exhaustive search for literature and conducted our systematic review in accordance to the highest reporting standards, our review is not without limitations. Firstly, studies evaluating differences in urban versus rural settings within subgroup analyses may not have been easily identified. Second, as in any systematic review, the findings are limited by the quality of the individual studies. Potential limitations included reporting bias [32,33,40,41,45,49,51,54,71,72], selection bias [9,35,54], lack of generalizability [9,28,36,37,39,50,51,59,71,73,74], limited sample size [28,36,37,39,50,64,73], no adjustment for confounders [8,28,35-37,40,50,54,64,74,75], and poor reporting (STROBE score below the 25th percentile) [8,28,36,37,40,44,50-52,54,59,64,70,71,74]. Third, we accepted a broad range of definitions for rural and urban populations, which may have affected study results. In 25 studies [8,28,34,36,37,40-43,45,46,50,52,53,55-60,64-66,72,74] no clear definition of rural and urban were provided and various definitions were used in the remaining studies. It is possible some of the heterogeneity observed in our pooled analysis is related to these differences in definitions, although subgroup analysis by studies with defined and undefined rural populations showed similar results. Fourth, between-study heterogeneity was high and not fully explained despite multiple subgroup analyses by setting, drug class, disease, age, country, publication year, and data source (self-report, administrative or medical records). Moreover, clinical indications and classification of disease may also have changed over time increasing the heterogeneity between studies. Last, our review only included adults with established cardiovascular disease as medication utilization for secondary prevention was expected to be high within patients; thus, improving the power to detect differences if differences exist between rural and urban patients. Moreover, as cardiovascular medications are widely prescribed medications in the general adult population, our results would be expected to be highly generalizable.

## Conclusions

In conclusion, we found no consistent relationship between rural versus urban residence and utilization of, or adherence with evidence-based cardiovascular medications among adults with cardiovascular disease or diabetes. There was substantial between-study variation that was only partially explained by the setting, age, and disease. Higher quality evidence is needed to determine if differences in cardiovascular medication utilization and adherence between urban and rural patients truly exist.

## Competing interests

The authors declare that they have no competing interests.

## Authors’ contributions

GKM and DLW screened, extracted and assessed the quality of studies. GKM conducted the meta-analysis. LT conducted the literature search and prepared the bibliography. GKM, DTE and FAM drafted the report. All authors had full access to all of the data (including statistical reports and tables) in the study and can take responsibility for the integrity of the data and accuracy of the data analysis. All authors read and approved the final manuscript.

## Pre-publication history

The pre-publication history for this paper can be accessed here:

http://www.biomedcentral.com/1471-2458/14/544/prepub

## Supplementary Material

Additional file 1 **The supplementary material includes Tables S1-S4, the literature search strategy, summary of the included study characteristics and results, and the supplementary meta-analyses.** Click here for file
