# Supplementary material for: Cardiovascular medication utilization and adherence among adults living in rural and urban areas: a systematic review and meta-analysis
Source: BMC Public Health. 2014 Jun 2;14:544. doi: 10.1186/1471-2458-14-544 (PMC4064809; doi:10.1186/1471-2458-14-544)
Supplement: Additional file 1 — The supplementary material includes Tables S1-S4, the literature search strategy, summary of the included study characteristics and results, and the supplementary meta-analyses. [file 1471-2458-14-544-S1.docx]

**Table S1: Literature search strategy - Medline**

| Medline and Medline In-Process (Licensed Ovid Interface) 1950 to Apr Week 2 2012  1. diabetes mellitus/  2. Diabetes Mellitus, Type 2/  3. ((diabet* or DM) adj5 ("type 2" or "type ii" or non insulin dependent or matur* onset or late onset)).ti,ab.  4. (diabet* not (juvenile or "type 1")).ti.  5. (diabetes adj5 (complication* or education)).mp.  6. (niddm or mody or T2DM).ti,ab.  7. (diabet* not gestational).ti.  8. or/1-7  9. exp Cardiovascular Diseases/  10. exp Hyperlipidemias/  11. ((cardiovascular or coronary or heart or cardia* or cardio* or myocardi* or pericardi*) adj3 disease).ti,ab.  12. or/9-11  13. 8 or 12  14. rural population/ or suburban population/ or urban population/  15. rural health/ or suburban health/ or urban health/  16. rural health services/ or suburban health services/ or urban health services/  17. "Catchment Area (Health)"/  18. (rural* or urban or semi-rural or semi-urban or city or non-urban or farm* or agricultur* or town* or village* or metropolitan or non-metropolitan or suburb* or remote or region or residence or jurisdiction or geographic or geography or location or residence or catchment).ti,ab.  19. (community adj (dwelling or resid* or based)).ti,ab.  20. (care adj network).ti,ab.  21. or/14-20  22. exp Drug Utilization/  23. Pharmacies/ut [Utilization]  24. exp Prescriptions/  25. exp Patient Compliance/  26. or/22-25  27. (Drug or drugs or prescrib* or prescription* or medication* or medicine or pharmac* or agent* or inhibitor* or intravenous or oral).ti,ab.  28. dt.fs.  29. exp Pharmaceutical Preparations/  30. or/27-29  31. exp "Physician's practice patterns"/  32. (adherence or compliance or concordance or usage or utilisation or utilization or underutili* or consumption or nonadherence or uptake).ti,ab.  33. ut.fs.  34. or/31-33  35. 30 and 34  36. ((Drug or drugs or prescrib* or prescription* or medication* or medicine or pharmac*) adj4 use*).ti,ab.  37. or/26,35-36  38. 13 and 21 and 37  39. animals/ not humans/  40. 38 not 39  41. (exp child/ or exp adolescent/ or exp infant/) not exp adult/  42. 40 not 41 |
| --- |

**Table S2. Study characteristics**

| **Author, year** | **Study design** | **Population** | **Country** | **Total N†** | **Setting (primary data source)** | **Age (y), mean (SD/SE)‡** | **Female (%)‡** | **Rural (%)‡** | **STROBE score§** |
| --- | --- | --- | --- | --- | --- | --- | --- | --- | --- |
| de Oliveira-Martins 2011[[38](#_ENREF_38)] | cross-sectional | HTN | Portugal | 1,042 | community pharmacy (patient interview) | 53.7 (SD 7.1) | 59 | 24 | 19 |
| Funkhouser 2011[[47](#_ENREF_47)] | cohort | AMI | US | 1,901 | ambulatory care practice (medical chart) | <65y: 13%  65-74y: 34%  >74y: 53% | 41 | 30 | 20 |
| Maio 2011(unpublished data V. Maio 2012)[[56](#_ENREF_56)] | cohort | AMI | Italy | 24,367 | population sample (administrative data) | 70.8 (SD 13.0) | 36 | 32 | 19.5 |
| Strom 2011[[69](#_ENREF_69)] | cross-sectional | DM | US | 52,817 | population sample (BRFSS) | 18-34y: 6%  35-49y: 19%  50-64y: 38%  >=65y: 38% | 50 | 21 | 19 |
| Yusuf 2011[[75](#_ENREF_75)] | cohort | CAD | Canada, Sweden, United Arab Emirates | 16,073 | community (patient interview) | 52.7 (SD 9.4) | NR | 28 | 20 |
| Ambardekar 2010[[27](#_ENREF_27)] | cross-sectional | CAD | US | 352,034 | hospital (Get With the Guidelines CAD Quality Improvement Program) | rural: 67.4 urban: 66.3 | rural: 43 urban: 42 | 6 | 19 |
| Asghari 2010[[29](#_ENREF_29)] | cohort | DM | Canada | 170,381 | population sample (administrative data) | 62 (SD 14) | 52 | 22 | 17.5 |
| Baldwin 2010[[30](#_ENREF_30)] | cross-sectional | AMI | US | 21,616 | hospital (medical chart) | 77.3 (SE 0.07) | 49 | 25 | 19.5 |
| DiMartino 2010[[42](#_ENREF_42)] | cohort | HF | US | 2,689 | Community dwelling (MCBS) | 79 (SE 0.2) | 56 | 28 | 19 |
| Ellis 2010[[43](#_ENREF_43)] | cross-sectional | HTN | US | 45,024 | population sample (BRFSS) | 18-34y: 6%  35-49y: 20%  50-64y: 37%  >=65y: 37% | rural: 52 urban: 52 | 37 | 20 |
| Friedman 2010[[46](#_ENREF_46)] | cohort | HTN | Canada | 207,473 | population sample (administrative data) | 66-70y: 40%  71-75y: 28%  76-80y: 18%  81-85y: 9%  85+ y: 5% | 58 | 14 | 19 |
| Hicks 2010[[51](#_ENREF_51)] | cross-sectional | DM with HTN | US | 778 | ambulatory care practice (physician survey) | rural: 58.3 (SE 1.2)  urban: 56.1 (SE 0.9)* | rural: 63 urban: 63 | 38 | 14.5 |
| Vanasse 2010[[2](#_ENREF_2)] | cohort | AMI | Canada | 44,806 | population sample (administrative data) | 66.5 (SD NR) | 35 | 25 | 18.5 |
| Wu 2010[[73](#_ENREF_73)] | cohort | HF | US | 136 | ambulatory care practice (medical chart, patient interview) | rural: 60 (SD 11)  urban: 62 (12) | rural: 25 urban: 39 | 64 | 20.5 |
| Fonarow 2009[[45](#_ENREF_45)] | repeat cross-sectional | AMI | US | 996,364 | hospital (NRMI) | 68.1 (SD 13.7) | 40 | hospitals: 8 | 18.5 |
| Niska 2009a[[61](#_ENREF_61)] | cross-sectional | CVD, DM, HTN, other | US | 4,964 | ambulatory care practice (NAMCS, NHAMCS) | 55-64y: 45%  65-80y: 55% | 56 | 20 | 17 |
| Niska 2009b[[62](#_ENREF_62)] | cross-sectional | AF | US | 1,771 | ambulatory care practice (NAMCS, NHAMCS) | <65y: 25%  65-75y: 29%  >75y: 46% | 49 | 14 | 17 |
| Goldman 2008[[49](#_ENREF_49)] | cross-sectional | AMI, HF | US | 2847 hospitals | hospital (Hospital Compare data) | NR | NR | hospitals: 38 | 20 |
| Ma 2008[[55](#_ENREF_55)] | cross-sectional | HTN | US | 50,574 | ambulatory care practice (NAMCS) | NR | NR | NR | 18 |
| Wan 2008[[71](#_ENREF_71)] | repeat cross-sectional | DM | Australia | 6,305 | ambulatory care practice (CARDIAB registry) | rural: 64 urban: 60 | NR | 54 | 16 |
| Williams 2008[[72](#_ENREF_72)] | cross-sectional | AMI, HF | US | 3,138 hospitals | hospital (Joint Commission performance indicator data) | NR | NR | hospitals: 30 | 17.5 |
| Clark 2007[[35](#_ENREF_35)] | cross-sectional | HF | Australia | 22,060 | ambulatory care practice (CASE study) | NR | NR | 29 | 16.5 |
| Colleran 2007[[36](#_ENREF_36)] | cohort | CVD | US | 200 | ambulatory care practice (medical chart) | range  rural: 50-82y urban: 52-74y | rural: 40 urban: 55 | 50 | 15 |
| Lutfiyya 2007[[54](#_ENREF_54)] | cross-sectional | AMI, HF | US | 4,203 hospitals | hospital (Hospital Compare data) | NR | NR | hospitals: 11 | 13.5 |
| Perez-Fernandez 2007[[64](#_ENREF_64)] | cross-sectional | HTN | Spain | 2,884 | population sample (patient survey) | 41.6 (SD 15.3) | 54 | 45 | 16 |
| Rowan 2007[[67](#_ENREF_67)] | cross-sectional | AF | US | NR | ambulatory care practice (NAMCS) | 18-59y: 11%  60-75y: 37%  >75y: 52% | 49 | 23 | 17 |
| Byrne 2006[[34](#_ENREF_34)] | cross-sectional | CAD | Ireland | 1,611 | ambulatory care practice (medical chart) | 66 (SD 9.1) | 35 | NR | 20 |
| Czarnecka 2006[[37](#_ENREF_37)] | cross-sectional | HTN | Poland | 222 | ambulatory care practice (patient survey) | 56.9 y (SD 8.6)* | 55 | 20 | 8.5 |
| DeWilde 2006[[41](#_ENREF_41)] | repeat cross-sectional | AF | UK | 12,267 | ambulatory care practice (DIN-LINK data) | 35-64y: 16%  65-74y:25%  75-84y: 39%  ≥85y: 20% | 47 | NR | 16.5 |
| Tuesca-Molina 2006[[70](#_ENREF_70)] | cross-sectional | HTN | Spain | 1,719 | population sample (patient survey) | 60-69y: 42%  70-79y: 39%  80+y: 19% | 63 | 6 | 15 |
| Bradley 2005[[33](#_ENREF_33)] | cross-sectional | AMI | US | 60,363 | hospital (NRMI) | 67.7 (SD 13.9) | 39 | hospitals: 17 | 20 |
| Nguyen 2005[[60](#_ENREF_60)] | cross-sectional | DM | US | NR | ambulatory care practice (NAMCS) | 30-44y: 10%  45-59y: 30%  60-74y: 40%  >74y: 21% | 53 | 21 | 17 |
| Rice 2005[[66](#_ENREF_66)] | cross-sectional | cardiac | US | 2,121 | population sample (CHI survey) | 18-24y: 4%  25-34y: 9%  35-44y: 16%  45-54y:32%  55-64y: 38% | 48 | 14 | 18.5 |
|  |  | HTN | US | 8,243 | population sample (CHI survey) | 18-24y: 5%  25-34y: 12%  35-44y: 22%  45-54y:32%  55-64y: 29% | 47 | 14 | 18.5 |
| Yiannakopoulou 2005[[74](#_ENREF_74)] | cross-sectional | HTN | Greece | 1,000 | hospital (patient survey) | 58.5 (SD 11.3) | 45 | 28 | 13 |
| Andrus 2004[[28](#_ENREF_28)] | cohort | DM | US | 187 | ambulatory care practice (medical chart) | rural: 55.1 (SD 13.7)  urban: 65.8 (SD 12.3)* | rural: 64 urban: 60 | 42 | 13 |
| Baldwin 2004[[31](#_ENREF_31)] | cross-sectional | AMI | US | 135,759 (4,085 hospitals) | hospital (medical chart) | 76.6 (SD 7.4) | 49 | 25 | 20.5 |
| Bradley 2004[[32](#_ENREF_32)] | repeat cross-sectional | AMI | US | 335,244 | hospital (NRMI) | 67.1 (SD 13.9) | 38 | hospitals: 18 | 20.5 |
| Ko 2004[[53](#_ENREF_53)] | cohort | CVD, DM | Canada | 396,077 | population sample (administrative data) | median 72 y | 55 | 18 | 19.5 |
| Pittrow 2004[[65](#_ENREF_65)] | cross-sectional | HTN | Germany | 17,485 | ambulatory care practice (physician survey) | 63.2 (SD 12.4) | 57 | NR | 17 |
| Psaltopoulou 2004[[9](#_ENREF_9)] | cross-sectional | HTN | Greece | 26,913 | community (patient interview) | 25-44y: 10%,  45-64y: 49%,  >=65y: 41% | rural: 62 urban: 52 | 62 | 19.5 |
| DeWilde 2003[[40](#_ENREF_40)] | repeat cross-sectional | CAD | UK | 30,448 | ambulatory care practice (DIN) | 35-44y: 1%  45-54y: 8%  55-64y: 21%  65-74y: 37%  75-84y: 33% | 41 | practices: 18 | 15 |
| Huttin 2002[[52](#_ENREF_52)] | cross-sectional | HTN | US | 1,844 | ambulatory care practice (NAMCS) | ≤44y: 11%  45-54y: 17%  55-64y: 21%  65-74: 27%  75-84y: 20%  >85y: 5% | 56 | NR | 13.5 |
| Majumdar 2001[[58](#_ENREF_58)] | cross-sectional | AMI | US | 5,138 | hospital (medical chart) | 67 (SD 14)* | 38 | 18 | 21 |
| Sheikh 2001[[68](#_ENREF_68)] | cohort | AMI | US | 2,285 | hospital (medical chart) | rural: 78.2  semi-rural: 76.6  urban: 74.1 | rural:43  semi-rural: 46  urban: 43 | 20 | 17 |
| Obisesan 2000[[63](#_ENREF_63)] | cross-sectional | HTN | US | 6,278 | population sample (NHANES III) | NR | NR | NR | 16.5 |
| Dellasega 1999[[39](#_ENREF_39)] | cohort | cardiac | US | 32 | hospital (patient survey) | rural: 73.3 (SD 4.6)  urban: 73.2 (SD 6.3) | rural: 33 urban: 24 | 47 | 18 |
| Flaker 1999 (Gage 2000)[[44](#_ENREF_44), [48](#_ENREF_48)] | cross-sectional | AF | US | 597 | hospital (medical chart) | rural: 80.7 (SD 7.6)  urban: 79.6 (SD 8.3)* | rural: 58 urban:54 | 26 | 16 |
| Majumdar 1999[[57](#_ENREF_57)] | cross-sectional | AMI | US | 622 | hospital (medical chart) | 66.4 | 37 | 27 | 20 |
| Banegas 1998[[8](#_ENREF_8)] | cross-sectional | HTN | Spain | 2,021 | community (patient interview) | NR | NR | NR | 12.5 |
| Munschauer 1997[[59](#_ENREF_59)] | cohort | AF | US | 651 | hospital (medical chart) | NR | NR | hospitals: 50 | 13 |
| Hense 1990[[50](#_ENREF_50)] | cohort | HTN | Germany | 289 | community dwelling sample (patient survey) | 30-49y: 24%  50-64y: 76% | 57 | 54 | 14 |
| AF=atrial fibrillation; AMI=acute myocardial infarction; BRFSS=Behavioral Risk Factor Surveillance System: CAD=coronary artery disease; CASE=Cardiac Awareness Survey and Evaluation study; CHI=California Health Insurance; CVD=cardiovascular disease; DM=diabetes; DIN=Doctor's Independent Network database; HF=heart failure; HTN=hypertension; MCBS=Medicare Current Beneficiary Survey: MEPS=Medical Expenditure Panel Survey: NAMCS=National Ambulatory Medical Care Survey; NHAMCS=National Hospital Ambulatory Medical Care Survey: NHANES=National Health and Nutritional Examination Survey; NR=not reported; NRMI=National Registry of Myocardial Infarction; SD=standard deviation; SE=standard error; y=years | | | | | | | | | |
| †All patients included in study; ‡When available, the age, sex and proportion rural data were reported for the subset of patients most relevant to this report’s outcomes (e.g. the subset with DM or CVD) §Studies were given 1 point for complete reporting and 0.5 points for partial reporting of items listed on the STROBE checklist (total possible points = 22); *Assumed to be SD or SE: measure of variance was not reported clearly by study authors. | | | | | | | | | |

**Table S3. Study results**

| **Author, year** | **Setting** | **Population** | **Outcome** | **Total N** | **Rural** | **Urban** | **Rural/urban unadjusted**  **OR (95% CI) †** | **Rural/urban adjusted**  **OR (95% CI)†** |
| --- | --- | --- | --- | --- | --- | --- | --- | --- |
| **Medication adherence or persistence** | | | | | | | | |
| Asghari 2010[[29](#_ENREF_29)] | community | DM | Regular ASA user (MPR ≥ 0.8) | 47,829 | 67.7% | 63.0% | 1.24 (1.18, 1.29)* | 1.14 (1.10, 1.18) |
|  |  |  | Regular ACEI/ARB user (MPR ≥ 0.8) | 76,482 | 73.5% | 70.5% | 1.16 (1.12, 1.21)* | 1.18 (1.14, 1.23) |
| Friedman 2010[[46](#_ENREF_46)] | community | HTN | therapy persistence over 2 years (any HTN medication) | 206,603 | NR | NR | NR | 1.28 (1.25, 1.32) |
|  |  |  | drug class persistence over 2 years (same class of medication as initial therapy) over 2 years | 206,603 | NR | NR | NR | 1.27 (1.23, 1.30) |
|  |  |  | MPR ≥ 0.8 over 2 years | 136,673 | NR | NR | NR | 1.22 (1.14, 1.32) |
| Wu 2010[[73](#_ENREF_73)] | amb. care practice | HF | medication adherence over 3 mo (% doses taken) | 136 | 91.6%  (SD 11.3) | 83.4% (20.4) | p=0.011 (data NR) | NR |
| Czarnecka 2006[[37](#_ENREF_37)] | amb. care practice | HTN | regular users of meds | 222 | 25.0% | 66.3% | 0.17 (0.08, 0.36)* | NR |
| Tuesca-Molina 2006[[70](#_ENREF_70)] | community | HTN | adherent to HTN medications: men | 530 | NR | NR | 5.36 (1.57, 18.23) | 3.98 (1.13, 13.93) |
|  |  |  | adherent to HTN medications: women | 905 | NR | NR | 1.01 (0.58, 1.77) | NR |
| Yiannakopoulou 2005[[74](#_ENREF_74)] | hospital | HTN | compliant with HTN meds | 1,000 | 10.0% | 16.90% | p<0.01 (data NR) | NR |
| Hense 1990[[50](#_ENREF_50)] | community | HTN | treatment persistence (identical medication at baseline and follow-up) | 204 | 55.0% | 55.9% | 0.96 (0.55, 1.67)* | NR |
| **Medication utilization** | | | | | | | | |
| de Oliveira-Martins 2011[[38](#_ENREF_38)] | community | HTN | treated for HTN | 571 | NR | NR | 0.51 (0.34, 0.75) | 0.46 (0.30, 0.72) |
| Funkhouser 2011[[47](#_ENREF_47)] | amb. care practice | AMI | prescribed BB (no contraindications) | 1,901 | 74.7% | 66.7% | 1.33 (1.12, 1.72) | 1.72 (1.31, 2.48) |
| Maio 2011 (unpublished data V. Maio 2012)[[56](#_ENREF_56)] | community | AMI | initiated on BB (no contraindications) | 24,367 | 68.4% | 67.3% | 1.08 (1.02, 1.15)* | rural (hill): 1.05 (0.91, 1.20),  rural (mountain): 0.92 (0.80, 1.04) |
| Strom 2011[[69](#_ENREF_69)] | community | DM | ASA user (weighted %) | 52,817 | 55.7% | 53.8% | p=0.15 (data NR) | 1.08 (0.96, 1.21) |
| Yusuf 2011[[75](#_ENREF_75)] | community | CAD | antiplatelet drug user | 669 | 64.2% | 64.1% | 1.00 (0.69, 1.45)* | NR |
|  |  |  | taking any HTN drug (ACE, ARB, BB, CCB, diuretic) |  | 77.2% | 78.7% | 0.91 (0.60, 1.40)* | NR |
|  |  |  | statin user |  | 64.8% | 72.8% | 0.69 (0.47, 1.00)* | NR |
| Ambardekar 2010[[27](#_ENREF_27)] | hospital | CAD | ASA at discharge | 352,034 | 90.5% | 95.0% | 0.58 (0.45, 0.75) | 0.80 (0.56, 1.16) |
|  |  |  | ACEI/ARB at discharge |  | 82.4% | 81.3% | 1.02 (0.82, 1.26) | 1.25 (1.03, 1.53) |
|  |  |  | BB at discharge |  | 86.2% | 91.3% | 0.62 (0.47, 0.83) | 0.96 (0.69, 1.33) |
|  |  |  | LLD at discharge |  | 83.4% | 86.5% | 0.60 (0.43, 0.84) | 1.12 (0.83, 1.52) |
| Asghari 2010[[29](#_ENREF_29)] | community | DM | ASA user | 170,381 | 31.0% | 27.0% | 1.21 (1.18, 1.25)* | 1.26 (1.22, 1.29) |
|  |  |  | ACEI/ARB user |  | 49.0% | 44.0% | 1.22 (1.20, 1.25)* | 1.29 (1.26, 1.32) |
| Baldwin 2010[[30](#_ENREF_30)] | hospital | AMI | ASA on discharge (no contraindications, weighted N, %) | 68,343 | Large: 78.0%,  Small: 77.4%,  Isolated: 64.7% | 82.0% | Large: RR 0.95 (p = NS), Small: RR 0.94 (p<=0.05), Isolated: RR 0.79 (p <=0.001) | Large: RR 0.97 (0.93, 1.00), Small: RR 0.99 (0.94, 1.02), Isolated: RR 0.84 (0.73, 0.93) |
|  |  |  | ACEI at discharge (weighted N, %) | 30,011 | Large: 62.7%,  Small: 62.6%,  Isolated: 68.8% | 61.2% | Large: RR 1.03 (p = NS), Small: RR 1.02 (p = NS), Isolated: RR 1.12 (p = NS) | Large: RR 1.05 (0.96, 1.13), Small: RR 1.04 (0.90, 1.17), Isolated: RR 1.16 (0.85, 1.37) |
|  |  |  | BB on discharge (no contraindications, weighted N, %) | 86,233 | Large: 68.3%,  Small: 59.9%,  Isolated: 53.4% | 69.2% | Large: RR 0.99 (p = NS), Small: RR 0.87 (p<=0.001), Isolated: RR 0.77 (p <=0.001) | Large: RR 1.01 (0.97, 1.05), Small: RR 0.92 (0.85, 0.98), Isolated: RR 0.82 (0.69, 0.94) |
| DiMartino 2010[[42](#_ENREF_42)] | community | HF | ACEI/ARB user | 2,689 | NR | NR | NR | 1.18 (0.98, 1.41) |
|  |  |  | BB user |  | NR | NR | NR | 1.04 (0.86, 1.27) |
| Ellis 2010[[43](#_ENREF_43)] | community | HTN | HTN medication user: Caucasian race | 38,268 | 87.9% | 87.1% | 1.08 (1.01, 1.15)* | 1.15 (0.93, 1.42) |
|  |  |  | HTN medication user: Black race | 6,756 | 89.5% | 88.9% | 1.07 (0.91, 1.25)* | NR |
| Friedman 2010[[46](#_ENREF_46)] | community | HTN | ACEI user | 95,773 | 47.1% | 46.0% | 1.04 (1.02, 1.07)* | NR |
|  |  |  | ARB user | 9,452 | 3.5% | 4.7% | 0.74 (0.69, 0.79)* | NR |
|  |  |  | BB user | 21,973 | 12.2% | 10.3% | 1.21 (1.16, 1.26)* | NR |
|  |  |  | CCB user | 23,603 | 10.3% | 11.6% | 0.88 (0.84, 0.91)* | NR |
|  |  |  | diuretic user | 56,672 | 32.9% | 26.4% | 1.36 (1.33, 1.40)* | NR |
| Hicks 2010[[51](#_ENREF_51)] | amb. care practice | DM, HTN | prescribe HTN medication or increase HTN dose for uncontrolled HTN | 478 | 21.1% | 33.2% | 0.54 (0.35, 0.83)* | NR |
| Vanasse 2010[[2](#_ENREF_2)] | community | AMI | ASA user | NR | NR | 86.3% | NR | multiple urban and rural groups; RR [p-value 99% CI] for urban (CA), rural (strong MIZ), (mod MIZ), (weak MIZ), (no MIZ), reference urban (CMA): 1.01 [NS], 1.03 [p<0.001], 1.03 [p<0.001], 0.98 [NS], 0.97 [NS] |
|  |  |  | ACEI user | NR | NR | 74.7% | NR | RR 1.00 [NS], 1.02 [NS], 1.05 [p<0.0001], 1.03 [NS], 1.04 [NS] |
|  |  |  | BB user | NR | NR | 81.2% | NR | RR 0.98 [NS], 1.02 [NS], 1.00 [NS], 0.95 [p<0.01], 0.97 [NS] |
|  |  |  | statin user | NR | NR | 72.1% | NR | RR 0.99 [NS], 1.02 [NS], 1.03 [NS], 1.02 [NS], 1.03 [NS] |
| Wu 2010[[73](#_ENREF_73)] | amb. care practice | HF | ACEI user (at baseline) | 136 | 80.5% | 55.1% | 3.36 (1.55, 7.27)* | NR |
|  |  |  | BB user (at baseline) |  | 89.7% | 87.8% | 1.21 (0.40, 3.63)* | NR |
| Fonarow 2009[[45](#_ENREF_45)] | hospital | AMI | LLD at discharge | 996,364 | NR | NR | NR | 0.88 (0.86, 0.89) |
| Niska 2009a[[61](#_ENREF_61)] | amb. care practice | CVD, DM, HTN, other | prescribed statin at physician visit (weighted %) | 4,964 | 36.2% (95% CI 29.7, 43.4%) | 38.1% (35.2, 41.1%) | p=0.64 (data NR) | 0.96 (0.70, 1.31) |
| Niska 2009b[[62](#_ENREF_62)] | amb. care practice | AF | prescribed warfarin at physician visit (no contraindications, weighted %) | 1,771 | 50.4% (95% CI 39.8, 61.0%) | 52.6% (48.4, 56.7%) | NS (data NR) | 1.02 (0.61, 1.70) |
| Goldman 2008[[49](#_ENREF_49)] | hospital | AMI | mean % difference in ASA at discharge stratified by bed size: <31 beds | 2573 hospitals |  |  | NR | -6.13% (95% CI -9.81, -2.43) |
|  |  |  | 31 to <65 beds |  |  |  | NR | -5.03% (-7.03, -3.02), |
|  |  |  | 65 to <120 beds |  |  |  | NR | -2.19% (-3.52, -0.86) |
|  |  |  | 120 to <240 beds |  |  |  | NR | -0.12% (-1.44, 1.20) |
|  |  |  | >=240 beds |  |  |  | NR | -2.86% (-4.90, -0.82) |
|  |  |  | mean % difference in BB at discharge stratified by bed size: <31 beds | 2302 hospitals |  |  | NR | -6.22% (-10.85, -1.59) |
|  |  |  | 31 to <65 beds |  |  |  | NR | -4.76% (-7.20, -2.32) |
|  |  |  | 65 to <120 beds |  |  |  | NR | -2.43% (-4.01, -0.84) |
|  |  |  | 120 to <240 beds |  |  |  | NR | 1.25% (-0.25, 2.76) |
|  |  |  | >=240 beds |  |  |  | NR | -0.59% (-3.06, 1.87) |
|  |  | HF | mean % difference in ACEI at discharge stratified by bed size: <31 beds | 2297 hospitals |  |  | NR | -1.58% (-8.71, 5.55) |
|  |  |  | 31 to <65 beds |  |  |  | NR | -4.07% (-7.82, -0.32) |
|  |  |  | 65 to <120 beds |  |  |  | NR | -2.43% (-4.96, 0.001) |
|  |  |  | 120 to <240 beds |  |  |  | NR | -0.02% (-2.33, 2.29) |
|  |  |  | >=240 beds |  |  |  | NR | 3.51% (-0.28, 7.31) |
| Ma 2008[[55](#_ENREF_55)] | amb. care practice | HTN | prescribed >=1 HTN medication at physician visit | 1,865 | 47.0% | 65.0% | 0.48 (0.37, 0.61)* | 1.89 (0.96, 3.70) |
| Wan 2008[[71](#_ENREF_71)] | amb. care practice | DM | HTN medication user (2002) | 3,219 | 34.0% | 26.5% | 1.43 (1.23, 1.66)* | NR |
|  |  |  | HTN medication user (2001) | 1,690 | 32.0% | 21.1% | 1.75 (1.40, 2.20)* | NR |
|  |  |  | HTN medication user (2000) | 1,396 | 28.8% | 14.1% | 2.46 (1.88, 3.21)* | NR |
|  |  |  | LLD user (2002) | 3,219 | 20.4% | 23.9% | 0.82 (0.70, 0.97)* | NR |
|  |  |  | LLD user (2001) | 1,690 | 24.8% | 18.4% | 1.46 (1.15, 1.86)* | NR |
|  |  |  | LLD user (2000) | 1,396 | 22.5% | 14.1% | 1.76 (1.34, 2.33)* | NR |
| Williams 2008[[72](#_ENREF_72)] | hospital | AMI | ASA on discharge (no contraindications) | 2881 hospitals | NR | NR | NR | 0.88 (0.79, 1.00) |
|  |  |  | ACEI/ARB at discharge (no contraindications) | 2709 hospitals | NR | NR | NR | 0.85 (0.76, 0.95) |
|  |  |  | BB at discharge (no contraindications) | 2887 hospitals | NR | NR | NR | 0.80 (0.71, 0.90) |
|  |  | HF | ACEI/ARB at discharge (no contraindications) | 3127 hospitals | NR | NR | NR | 0.88 (0.81, 0.96) |
| Clark 2007[[35](#_ENREF_35)] | amb. care practice | HF | ACEI user | 2,735 | 54.80% | 60.1% (95% CI 58, 62%) | 0.81 (0.68, 0.95)* | NR |
|  |  |  | loop diuretic user |  | 68.0% | 66.9% (65, 69%) | 1.05 (0.88, 1.26)* | NR |
|  |  |  | BB user |  | 11.5% | 11.8% (10, 13%) | 0.97 (0.75, 1.25)* | NR |
| Colleran 2007[[36](#_ENREF_36)] | amb. care practice | CVD | ASA/anti-platelet user | 200 | 47.0% | 93.0% | 0.07 (0.03, 0.16)* | NR |
|  |  |  | ACEI/ARB user |  | 55.0% | 77.0% | 0.37 (0.20, 0.67)* | NR |
|  |  |  | BB/CCB user |  | 51.0% | 88.0% | 0.14 (0.07, 0.29)* | NR |
|  |  |  | statin user |  | 42.0% | 87.0% | 0.11 (0.05, 0.22)* | NR |
| Lutfiyya 2007[[54](#_ENREF_54)] | hospital | AMI | weighted mean % per hospital receiving ASA at discharge (no contraindications) | NR | 82.0% | 93.9% | Mean difference: -11.9% (99% CI -4.4, -19.2%), p<0.01 | NR |
|  |  |  | weighted mean % per hospital receiving BB at discharge (no contraindications) | NR | 78.4% | 91.2% | -12.8% (99% CI -4.6, -20.8%), p<0.01 | NR |
|  |  | HF | weighted mean% per hospital receiving ACEI at discharge (no contraindications) | NR | 75.5% | 75.3% | 0.2% (99% CI -7.0, 7.4%) | NR |
| Perez-Fernandez 2007[[64](#_ENREF_64)] | community | HTN | HTN medication user | 372 | 72.7% (95% CI 66.2, 79.2%) | 71.4% (65.0, 77.8%) | 1.06 (0.68, 1.67)* | NR |
| Rowan 2007[[67](#_ENREF_67)] | amb. care practice | AF | warfarin user (weighted n, %) | 40,506,313 | 45.9% | 45.6% | p=0.94 (data NR) | NR |
| Byrne 2006[[34](#_ENREF_34)] | amb. care practice | CAD | prescribed ASA (no contraindications) | 1,587 | 82.0% | 80.0% | NS (data NR) | 0.95 (0.68, 1.33) |
|  |  |  | prescribed ACEI |  | 31.0% | 23.0% | p<0.01 (data NR) | 0.56 (0.44, 0.78) |
|  |  |  | prescribed LLD |  | 47.0% | 47.0% | NS (data NR) | 1.07 (0.81, 1.41) |
|  |  |  | prescribe nitrates |  | 46.0% | 55.0% | p<0.01 (data NR) | NR |
| DeWilde 2006 (unpublished data I. Carey 2011)[[41](#_ENREF_41)] | amb. care practice | AF | anticoagulant user | 9,399 | Town 48.0%;  Village 50.1% | 48.9% | Town: 0.96 (0.84, 1.10)*;  Village: 1.05 (0.93, 1.19)* | Town: 0.96 (0.76, 1.20);  Village: NR |
| Bradley 2005[[33](#_ENREF_33)] | hospital | AMI | BB at discharge | 60,363 | NR | NR | NR | NS (data NR) |
| Nguyen 2005[[60](#_ENREF_60)] | amb. care practice | DM | ASA user (no contraindications, weighted n, %) | 134 940 000 | 3.0% | 2.5% | NR | 1.23 (1.15, 1.33) |
| Rice 2005[[66](#_ENREF_66)] | community | CVD | taking medications to control heart disease | 2,114 | NR | NR | NR | 0.95 (0.71, 1.28) |
|  |  | HTN | HTN medication user | 8,217 | NR | NR | NR | 1.14 (0.97, 1.33) |
| Andrus 2004[[28](#_ENREF_28)] | amb. care practice | DM | ASA user | 187 | 17.9% | 39.4% | 0.34 (0.17, 0.67)* | NR |
| Baldwin 2004[[31](#_ENREF_31)] | hospital | AMI | ASA at discharge (no contraindications) | 43634 | Large: 74.4%,  Small: 71.1%,  Remote: 67.7% | 76.4% | p<=0.001 (data NR) | Large: RR 0.99 (0.96, 1.01),  Small: 0.95 (0.92, 0.98),  Remote: 0.90 (0.86, 0.96) |
|  |  |  | BB at discharge (no contraindications) | 10056 | Large: 49.6%,  Small: 47.2%,  Remote: 55.4% | 51.8% | p=0.16 (data NR) | Large: RR 0.97 (0.89, 1.05),  Small: 0.93 (0.83, 1.05),  Remote: 1.07 (0.83, 1.38) |
|  |  |  | ACEI at discharge | 12459 | Large: 63.1%,  Small: 56.9%,  Remote: 64.0% | 59.4% | p=0.021 (data NR) | Large: RR 1.06 (1.01, 1.11),  Small: 0.94, (0.87, 1.02),  Remote: 1.08 (0.93, 1.24) |
|  |  |  | avoid CCB at discharge | 6334 | Large: 84.7%,  Small: 86.2%,  Remote: 89.7% | 83.6% | p=0.27 (data NR) | Large: RR 1.01 (0.97, 1.05),  Small: 1.03 (0.98, 1.08),  Remote: 1.06 (0.99, 1.14) |
| Bradley 2004[[32](#_ENREF_32)] | hospital | AMI | BB at discharge (crude rate over study period, mean (SD)) | 335,244 | 56.2% (12.8) | 57.5% (12.2) | NR | NR |
|  |  |  | BB at discharge (adjusted rate over study period, mean (SD)) |  | 59.9% (NR) | 60.9% (NR) | NR | NR |
| Ko 2004[[53](#_ENREF_53)] | community | CVD, DM | prescribed statin | 388,845 | 15.9% | 19.9% | 0.76 (0.75, 0.78)* | NR |
| Pittrow 2004[[65](#_ENREF_65)] | amb. care practice | HTN | prescribed HTN medication | 17,485 | NR | NR | NR | NS (data NR) |
| Psaltopoulou 2004[[9](#_ENREF_9)] | community | HTN | HTN medication user | 6501 | NR | NR | NR | 1.34 (1.15, 1.57) |
| DeWilde 2003[[40](#_ENREF_40)] | amb. care practice | CAD | prescribed statin | 30,448 | 27.0% | 27.0% | p=0.79 (data NR) | NR |
| Huttin 2002[[52](#_ENREF_52)] | amb. care practice | HTN | HTN medication user | 1,884 | NR | NR | NR | 0.99 (0.80, 1.23) |
| Majumdar 2001[[58](#_ENREF_58)] | hospital | AMI | CCB at discharge | 5,138 | 11.8% | 17.2% | 0.65 (0.52, 0.80) | 0.98 (0.65, 1.49) |
| Sheikh 2001[[68](#_ENREF_68)] | hospital | AMI | ASA at discharge (no contraindications) | 643 | 68.7% | 81.5% | 0.50 (0.34, 0.73)* | NR |
|  |  |  | BB at discharge (no contraindications) | 199 | 32.4% | 37.0% | 0.82 (0.38, 1.74)* | NR |
| Obisesan 2000[[63](#_ENREF_63)] | community | HTN | Age adjusted prevalence of treatment for HTN: 40 to 59 years, Southern states | NR |  |  | NR | NR |
|  |  |  | Black women |  | 67.6% | 63.1% |  |  |
|  |  |  | Black men |  | 41.1% | 32.0% |  |  |
|  |  |  | White men |  | 35.6% | 53.0% |  |  |
|  |  |  | Age adjusted prevalence of treatment for HTN: 40 to 59 years, Non-Southern states | NR |  |  | NR | NR |
|  |  |  | White women |  | 59.1% | 74.5% |  |  |
|  |  |  | White men |  | 0.2% | 5.8% |  |  |
|  |  |  | Age adjusted prevalence of treatment for HTN: 60 to 79 years, Southern states | NR |  |  | NR | NR |
|  |  |  | Black women |  | 76.1% | 69.6% |  |  |
|  |  |  | Black men |  | 65.2% | 69.0% |  |  |
|  |  |  | White women |  | 66.5% | 57.6% |  |  |
|  |  |  | White men |  | 54.7% | 61.8% |  |  |
|  |  |  | Age adjusted prevalence of treatment for HTN:  60 to 79 years,  non-Southern states | NR |  |  | NR | NR |
|  |  |  | Black women |  | 71.0% | 70.7% |  |  |
|  |  |  | Black men |  | 52.2% | 55.1% |  |  |
|  |  |  | White women |  | 58.0% | 65.8% |  |  |
|  |  |  | White men |  | 62.5% | 45.5% |  |  |
| Dellasega 1999[[39](#_ENREF_39)] | hospital | CVD | CV medication user (at 20 weeks) | 32 | 87% | 76.5% | 2.00 (0.31, 12.89) | NR |
| Flaker 1999 (Gage 2000)[[44](#_ENREF_44), [48](#_ENREF_48)] | hospital | AF | antithrombotic at discharge (all patients) | 597 | 46.8% | 57.9% | 0.64 (0.45, 0.92) | 0.59 (0.40, 0.83) |
|  |  |  | antithrombotic at discharge (no contraindications) | 234 | 54.8% | 74.5% | 0.41 (0.23, 0.74) | NR |
| Majumdar 1999[[57](#_ENREF_57)] | hospital | AMI | LLD upon admission | 622 | 32.1% | 38.8% | 0.75 (0.51, 1.09) | p=NS (data NR) |
| Banegas 1998[[8](#_ENREF_8)] | community | HTN | HTN medication user (weighted) | NR | 69.1% (60.1, 77.1%) | 73.0% (67.5, 78.1%) | p=NS (data NR) | NR |
| Munschauer 1997[[59](#_ENREF_59)] | hospital | AF | antithrombotic at discharge | 651 | 43.0% | 64.0% | NR | p<0.0001 (data NR) |
| amb.=ambulatory; ACEI=angiotensin converting enzyme inhibitor; AF=atrial fibrillation; AMI=acute myocardial infarction; ARB=angiotensin receptor blocker; ASA=acetylsalicylic acid; BB=beta-blocker; CA=Census Agglomerations; CAD=coronary artery disease; CCB=calcium channel blocker; CMA=Census Metropolitan Area; CI=confidence interval; CV=cardiovascular; CVD=cardiovascular disease; DM=diabetes; HF=heart failure; HTN=hypertension; LLD=lipid lowering drug; MIZ=Metropolitan Influenced Zone; MPR=medication possession ratio; N=number; NR=not reported; NS=not statistically significant; OR=odds ratio; RR=relative risk; SD=standard deviation | | | | | | | | |
| † Data are OR (95% CI) unless specified otherwise; *Calculated using Review Manager software | | | | | | | | |

**Table S4. Supplementary meta-analyses of medication utilization outcomes**

| **Analysis** | **N**  **cohorts** | **Rural/urban OR of treatment (95% CI)** | **I^2^ (%)** | **Subgroup difference** |
| --- | --- | --- | --- | --- |
| **Cardiovascular medication utilization** | | | | |
| **Subgroup analysis – adjusted** | |  |  |  |
| *Drug class* |  |  |  |  |
| ASA, antithrombotic or anticoagulant | 9 | 1.00 (0.88, 1.14) | 88 | P=0.74 |
| ACEI or ARB | 6 | 0.98 (0.79, 1.22) | 96 |  |
| Other antihypertensive drug | 13 | 1.04 (0.92, 1.19) | 78 |  |
| Lipid lowering drug | 4 | 0.94 (0.83, 1.07) | 37 |  |
| *Healthcare system* |  |  |  |  |
| Non-universal system | 21 | 1.01 (0.92, 1.10) | 88 | P=0.43 |
| Universal | 2 | 1.13 (0.86, 1.49) | 84 |  |
| *Study quality* |  |  |  |  |
| Above median quality score | 13 | 1.04 (0.92, 1.17) | 87 | P=0.67 |
| Median or lower quality score | 10 | 1.00 (0.85, 1.16) | 94 |  |
| *Rural-urban definition* |  |  |  |  |
| No definition | 14 | 1.00 (0.92, 1.10) | 87 | P=0.73 |
| Rural and urban defined | 9 | 1.04 (0.89, 1.21) | 86 |  |
| **Sensitivity analysis – adjusted** | | | | |
| **Correlation value for pooling multiple outcomes within studies** | | | | |
| 0.25 | 23 | 1.02 (0.91, 1.13) | 97 | NA |
| 0.50 | 23 | 1.02 (0.91, 1.13) | 97 |  |
| 0.75 | 23 | 1.02 (0.91, 1.13) | 97 |  |
| 1.00 | 23 | 1.02 (0.91, 1.13) | 97 |  |
| ACEI=angiotensin converting enzyme inhibitor; ARB=angiotensin receptor blocker; CI=confidence interval; N=number of cohorts pooled; NA=not applicable; OR=odds ratio. | | | | |
